# Comparison of four DLL3 antibodies performance in high grade neuroendocrine lung tumor samples and cell cultures

**DOI:** 10.1186/s13000-019-0827-z

**Published:** 2019-05-20

**Authors:** Luka Brcic, Christian Kuchler, Sylvia Eidenhammer, Daniela Pabst, Franz Quehenberger, Adi F. Gazdar, Helmut Popper

**Affiliations:** 1 Diagnostic and Research Center for Molecular BioMedicine, Diagnostic and Research Institute of Pathology, Medical University of Graz, Neue Stiftingtalstrasse 6, 8010 Graz, Austria; 2 Hamon Center for Therapeutic Oncology Research and Department of Pathology, University of Texas Southwestern Medical Center, 6000 Harry Hines Blvd, Dallas, TX USA; 3 Institute for Medical Informatics, Statistics and Documentation, Medical University of Graz, Auenbruggerplatz 2, 8036 Graz, Austria

**Keywords:** Small cell carcinoma, Large cell neuroendocrine carcinoma, Cell culture, DLL3, TP53, RB1

## Abstract

**Background:**

Small cell lung cancer (SCLC) is usually diagnosed in the advanced stage. It has a very poor prognosis, with no advancements in therapy in the last few decades. A recent phase 1 clinical study, using an antibody-drug conjugate directed against DLL3, showed promising results. A prerequisite for this therapy is an immunohistochemical test for DLL3 expression. The antibody used in the clinical trial was bound to a specific platform, which is not available in all pathology laboratories. In this study, the expression of DLL3 was analyzed using different DLL3 antibodies in high-grade neuroendocrine tumors of the lung and cell cultures. Additionally, correlation of DLL3 expression with Rb1 loss and TP53 mutation was evaluated.

**Methods:**

The study cohort consisted of surgically resected cases, 24 SCLC and 29 large cell neuroendocrine carcinoma (LCNEC), from which tissue microarrays (TMAs) were constructed. The validation cohort included 46 SCLC samples, mostly small biopsies. Additionally, well-characterized SCLC cell lines were used. Immunohistochemical analysis was performed using four different DLL3 antibodies, as well as TP53 and Rb1 antibodies. Expression was evaluated microscopically and manually scored.

**Results:**

The comparison of all DLL3 antibodies showed poor results for the overall agreement, as well as positive and negative agreement. Differences were observed regardless of the applied cut-off values and the tumor type. The antibody used in the clinical trial was the only which always positively stained the tumor cells obtained from cell cultures with known DLL3 expression and was negative on cells that did not express DLL3. There was no correlation between p53 and DLL3 expression in SCLC and LCNEC. *RB1* loss in SCLC showed statistical significant correlation with the DLL3 positivity (*p* = 0.037), while no correlation was found in LCNEC.

**Conclusion:**

The DLL3 antibody used in the clinical trial demonstrated superiority in the detection of DLL3 expression. Cell cultures, which can be used for DLL3 antibodies as positive and negative probes, were established. Evidence of DLL3 expression in high proportions of patients with LCNEC might provide basis for studies of new therapy options in this group of patients.

## Introduction

Small cell lung carcinoma (SCLC) and large cell neuroendocrine carcinoma (LCNEC) are high-grade pulmonary neuroendocrine tumors, with a poor prognosis. Advances in therapy have not been significant in the last three decades [1]. Small cell lung carcinoma represents 13–15% of the primary lung malignancies [2] and occurs in the elderly population, almost exclusively in smokers, with a 5-year survival rate of 7% [3]. At the time of diagnosis, patients usually present with distant metastases; they receive the platinum-etoposide combination chemotherapy as the standard treatment regimen [4, 5]. Response to the therapy is very high, but so is the recurrence. The only approved second-line treatment is topotecan, which has a low response rate (5–24%), with an overall survival < 7 months [6]. The progress in therapy for adenocarcinoma and squamous cell carcinoma of the lung have been achieved by identifying the targetable molecular changes in the former, and by applying immune checkpoint inhibitors in both. The effectiveness of immune checkpoint inhibitors is less in SCLC, unlike non-small cell lung carcinomas (NSCLC). The programmed death-ligand 1 (PD-L1) is expressed only in a small percentage of SCLC. On the other hand, the most common genetic changes in SCLC, TP53 and RB1 mutations and amplifications of SOX2 and SRSF1 are not targetable as these are tumor suppressor gene mutations [7]. The two main therapeutic approaches used for more than three decades for LCNEC are the SCLC-like treatment and the non-neuroendocrine carcinomas-like treatment that is based on resection and/or platinum-based protocols. Recent reports have demonstrated the possibility of a gene expression-based therapy for LCNEC [8, 9]. LCNEC with RB1 mutations, as in SCLC, might best respond to an SCLC-like chemotherapy, whereas LCNEC tumors with PTEN loss and/or PI3KCA activating mutations might be optimally treated with platinum-based therapy [8, 9].

Recently, Saunders et al. were able to identify the overexpression of delta-like ligand 3 (DLL3) using tumor-initiating cells obtained from SCLC and LCNEC patient-derived xenografts [10]. The DLL3, normally expressed in fetal brain, plays a physiological role in development [11–13]. It is the only member of the Notch receptor ligand family that inhibits the Notch receptor pathway [10]. Notch inactivation is found in most of the SCLC cells, and correlates to the gene expression of the neuroendocrine markers [14]. Furthermore, the expression of DLL3 is closely related to neuroendocrine differentiation, and the expression of the transcription factor achaete-scute homolog 1 (ASCL1), which is an important oncogenic driver for SCLC [15], regulates growth and survival of the SCLC cells and is found in 75% of SCLC cells [16]. Some SCLC cells, which express POU class 2 homeobox 3 (POU2F3), do not present neuroendocrine features [17]. Saunders et al., demonstrated DLL3 protein expression by immunohistochemistry in 65% of LCNEC cells and 72% of treatment-naïve SCLC cells, whereas the normal lung parenchyma was completely negative [10]. Thus, DLL3 protein has emerged as very promising drug target.

A recent first-in-human, first-in-class, open-label phase 1 study using rovalpituzumab tesirine, an antibody-drug conjugate directed against DLL3, demonstrated significant antitumor activity in patients with SCLC [18], especially when they have high expression of DLL3. It has already been shown that the overall expression of DLL3 in SCLC is high [10, 19]. However, several DLL3 antibodies are available, but correlation studies are yet to be conducted. The antibody used in clinical trials is bound to a specific platform, which is not available in all pathology laboratories, so a search for the adequate substitution is necessary. Unlike the release of new drugs in the USA, which is usually accompanied by a companion test, the European Medicines Agency (EMA) allows use of any validated test. In other words, companies or pathology laboratories can develop their own test, which than has to be validated.

Our aim was to investigate four different antibodies for their reliability to detect DLL3 expression in high-grade neuroendocrine tumors of the lung and well characterized cell cultures. Furthermore, we aimed to analyze if there is a correlation between DLL3 expression with the TP53 mutation or Rb1 loss, which might aid in directing cases of LCNEC for a possible DLL3 staining and eventual treatment.

## Materials and methods

### Patient cohort

The study cohort consisted of chemo-naïve, surgically resected high-grade neuroendocrine tumors of the lung, obtained from the Lung Archive, Institute of Pathology, Medical University of Graz, from 1996 to 2012, with enough tumor tissue for adequate analysis. Altogether 53 tumors were selected, 24 small cell lung carcinomas and 29 large cell neuroendocrine carcinomas. For each tumor, all slides were re-evaluated to confirm the diagnosis and to select the most adequate block for a tissue microarray (TMA) construction. Each tumor was represented by several cores, each 1 mm in diameter. Additionally, one normal lung tissue core was used as control for each patient. Two TMAs were constructed. As these tissues were derived from resection specimen, heterogeneous expression pattern, if present, could be evaluated.

As a validation cohort for small cell lung cancer, 46 tumor samples, from chemo-naïve patients, were selected. In this group, 34 samples were small biopsies and 12 were resections. All available slides were re-evaluated and one representative block was chosen from each patient for immunohistochemical analysis. In the validation set the expression pattern on small biopsies could be evaluated.

Survival data for study cohorts were obtained from the Main Association of Austrian Social Security Institutions.

### Immunohistochemistry and scoring

From each TMA block and validation cohort sample, a 4-μm thick sections were cut and mounted on positively charged glass slides. Staining was performed with four different DLL3 antibodies. DLL3 ready-to-use assay (clone SP347, Ventana, Roche, Tucson, AZ, USA) (VenA), after pre-treatment with CC1 for 80 min, and using OptiView detection kit (Ventana) on Benchmark Ultra slide staining instrument (Ventana). Furthermore, three additional DLL3 antibodies were used: NBP2–24669 (1:150; Novus Biological, Littleton, CA, USA, (NovA)); PA5–26336 (1:150; Thermo Fisher Scientific, Waltham, MA, USA (TherA)); and ab103102 (1:500; Abcam, Cambridge, MA, USA (AbcA)). All three were stained on DAKO Autostainer (DAKO, Glostrup, Denmark), after pre-treatment with MW 9.0 for 40 min at 150 W. EnVision Kit 5007 (DAKO, Glostrup, Denmark), and DAB as the chromogen, were used for detection.

For each antibody, the DLL3 positive membranous and/or cytoplasmic reaction was evaluated microscopically and percentage of positive tumor cells, regardless of intensity, was scored manually for each core (by HP and LB). Afterwards, an average value per tumor was calculated. Discordant cases were reviewed together and a consensus was reached on all cases.

The cut-off values for the percentage of positive tumor cells were set on 25, 50 and 75%. Since in the clinical study all cases with DLL3 expression in more than 50% of tumor cells were regarded as high [18], we wanted to see if additional cut-offs might influence concordance of the antibodies, and/or relation with survival, p53 and Rb1.

Analysis of p53 was performed using DAKO ready to use antibody on Omnis platform (both DAKO, Glostrup, Denmark), using Envision Flex (DAKO, Glostrup, Denmark), with DAB as detection chromogen. Anti-Rb antibody (1F8, 1:100, Abcam, Cambridge, MA, USA) was used, stained on DAKO Autostainer (DAKO, Glostrup, Denmark), after pre-treatment with MW 9.0 for 40 min at 150 W. EnVision Kit 5007 (DAKO, Glostrup, Denmark), and DAB were used for detection.

Positive nuclear reaction was evaluated for both p53 and Rb1, and expressed as percentage of tumor cells.

### Cell cultures and immunocytochemistry

Different SCLC cell lines (NCI-H69, NCI-H735, NCI-H1048, NCI-H740, and NCI-H187) were purchased from American Type Culture Collection (ATCC, LGC Standards GmbH, Wesel, Germany) and cultivated in media recommended by ATCC. In addition, two sub-clones NCI-H69a and NCI-H69s, provided by Dr. A. Gazdar were previously characterized in his laboratory (Table 1). A neuroendocrine (NE) score was developed. Briefly, they used expression of 25 genes that had a strong positive correlation with neuroendocrine differentiation, and 25 genes having strong negative correlation with neuroendocrine differentiation; in other words, a 50-gene lung cancer-specific neuroendocrine signature was developed. Positive NE score correlated with neuroendocrine differentiation, and was present in most of the SCLC cells; while negative score correlated with the absence of neuroendocrine differentiation [20]. The NCI-H69a is an adherent cell line, negative for neuroendocrine markers and DLL3, whereas the NCI-H69s cell line grows in spheres, is positive for neuroendocrine markers and expresses DLL3 (Table 1).

**Table 1:** Expression of neuroendocrine markers in SCLC cell cultures.

	Neuroendocrine Correlation r	NCI-H187	NCI-H735	NCI-H740	NCI-H1048	NCI-H69s	NCI-H69a
NE score	1.00	0.9	0.6	0.9	0.0	0.9	-0.2
DLL3	0.65	5.715	4.414	3.622	1.36	3.797	1.857
NOTCH3	-0.63	1.174	1.641	0.164	2.869	0.933	4.038
NOTCH1	-0.37	1.143	3.157	0.73	3.474	1.249	2.382
ASCL1	0.49	8.319	8.625	7.792	1.656	6.895	1.103
POU2F3	-0.73	0.195	0.472	0.101	7.813	0.33	0.24

Legend: *NE score* neuroendocrine score

Cell lines were cultivated until confluence; cells were harvested and a small proportion was sedimented onto glass slides, followed by fixation with formalin for 10 min. As a test with alcohol fixation resulted in no staining of DLL3-positive cells, formalin fixation was also used in the cell cultures.

In addition, after harvesting, cells were centrifuged, a cell pellet fixed in 10% formalin, and embedded in paraffin block. In this way cell blocks of each cell line were produced, 4 μm-thick sections were cut and mounted on positively charged glass slides.

Immunocytochemistry for all above samples was performed using the same DLL3 antibodies, and applying the same protocols as stated above. Positive expression of DLL3 was evaluated under the microscope, and scored as described above.

### Statistical analysis

Data were represented as percentages and medians. Agreement between antibodies on DLL3 positivity was assessed by Cohen’s kappa. The agreement on percentage of positive cells was assessed by the Spearman correlation coefficient. The statistical software R 3.5.1 (www.r-project.org) was used for calculations. The significance threshold for statistical tests was *P* < 0.05.

## Results

### Comparison of the expression of DLL3 using 4 different DLL3 antibodies in TMA samples

**Fig. 1:**
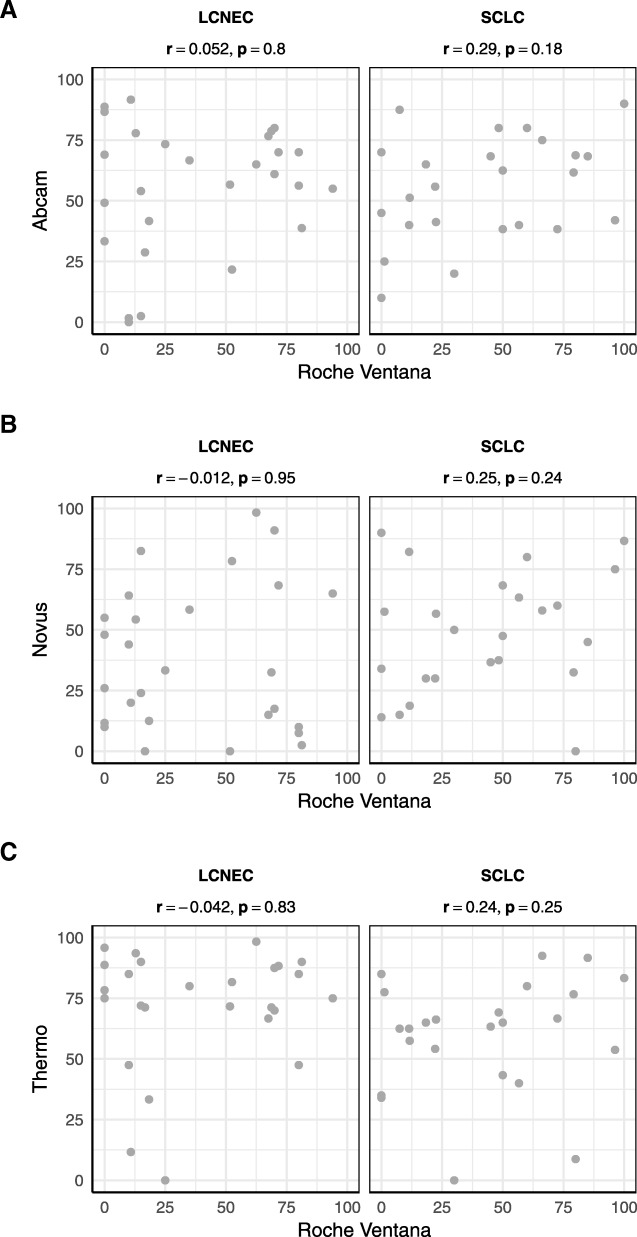
Presentation of correlation of VenA tumor cell positivity in SCLC and LCNEC, with AbcA (**a**), NovA (**b**) and TherA (**c**), none of which was significant

DLL3 positive reactions were observed in the SCLC as well as LCNEC samples. In the whole TMA cohort, NovA and VenA showed a lower intensity and a lower percentage of positive tumor cells, compared to AbcA and TherA. The same was true for separate subgroup analysis of SCLC and LCNEC tumors (Fig. 1a-c).

The analysis of the VenA-stained SCLC cells demonstrated that 20.8% (5/24) of the cases showed staining of ≥75% of tumor cells and 45.8% (11/24) cases showed positive reaction in ≥50% of tumor cells, whereas 58.3% (14/24) cases showed positivity in ≥25% of tumor cells. With NovA, TherA, and AbcA the distribution values for positively stained tumor cells were, ≥75%: 20.8% (5/24), 29.2% (7/24), 20.8% (5/24), ≥50%: 50.0%(12/24), 75.0% (18/24), 58.3% (14/24), and ≥ 25%: 83.3% (20/24) 91.7% (22/24), 91.7% (22/24), respectively.

For the LCNEC tumor cells, after applying the cut-off of ≥25% of positive tumor cells, the distribution values for positively stained tumor cells were 51.9% (14/27) with VenA, 55.6% (15/27) with NovA, 92.6% (25/27) with TherA and 85.7% (24/28) with AbcA.

Comparing VenA with the other antibodies, the overall agreement in percentage of positive tumor cells ranged from 45.8 to 83.3%. Positive agreement rose significantly with the applied cut-off value for positivity (≥25, ≥50, ≥75), while negative agreement was high below the cut-off of 25%, but gradually decreased with increase in cut-off value. Kappa values were extremely poor, ranging from − 23.2 to 33.9. These data were applicable to the whole TMA cohort as well as for the separate analysis of SCLC and LCNEC.

The percentage of positive tumor cells of all four antibodies did not show correlation with survival, in the whole group of analyzed samples, as well as in separate analyses for SCLC and LCNEC.

### Comparison of the expression of DLL3 using 4 different DLL3 antibodies in the validation SCLC cohort

**Fig. 2:**
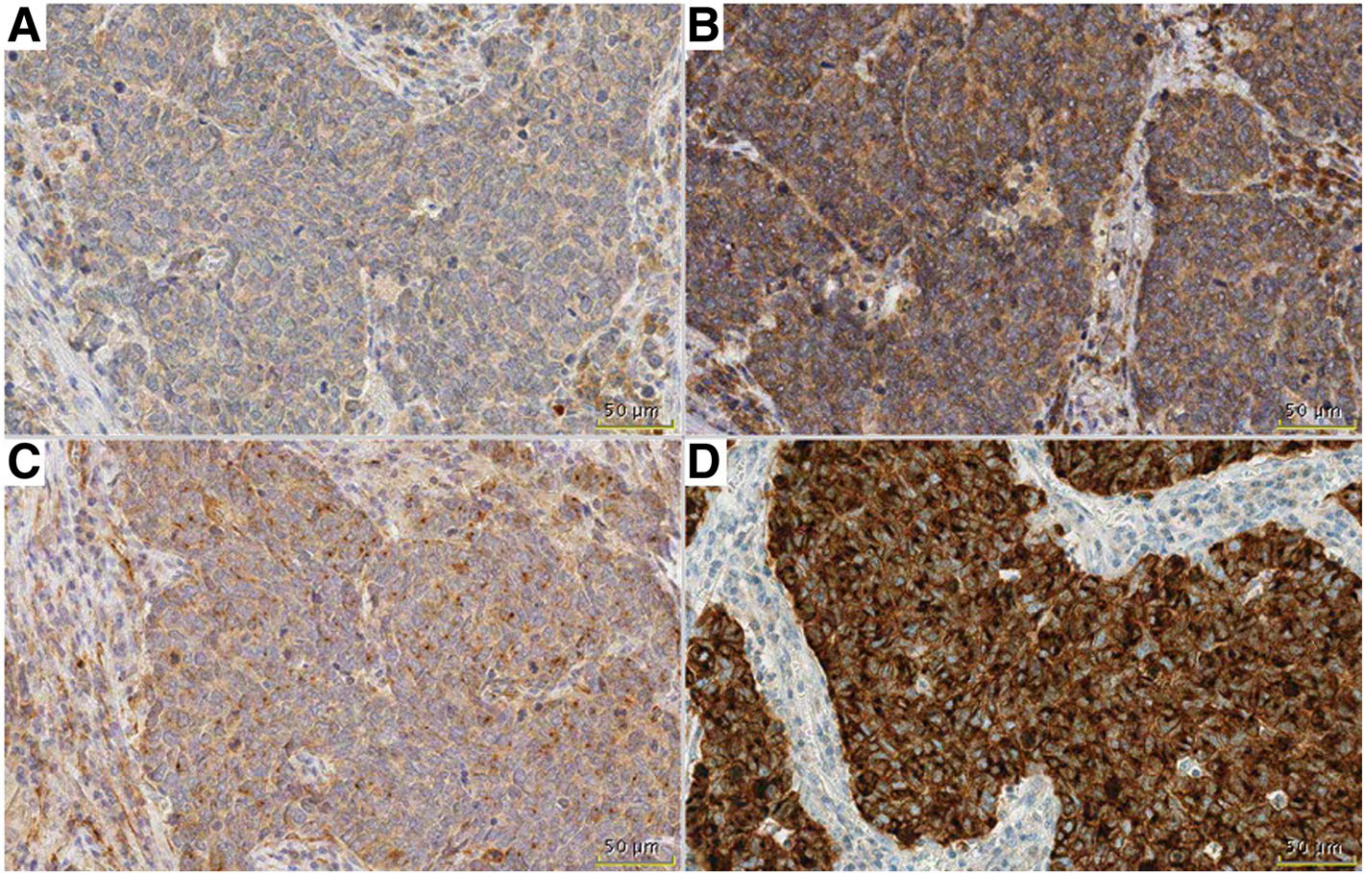
DLL3 staining in one sample in validation cohort, demonstrating difference between VenA (D) with strong staining intensity in all tumor cells, while all other antibodies (TherA-A, AbcA-B, NovA-C) are showing weaker intensity and positivity is not present in all tumor cells. Bar 50 μm

In the validation cohort, VenA and AbcA showed a higher intensity, as well as percentage of positive tumor cells, compared to NovA and TherA (Fig. 2). With VenA, 36.6% (15/41) of the cases showed positive reaction in ≥75% of tumor cells, compared to 28.3% (13/46) with AbcA. For ≥50%, VenA and AbcA showed 78.0% (32/41) and 69.6% (32/46), respectively, whereas the correlations for ≥25% were 82.9% (34/41) and 91.3% (42/46), respectively. The cut-offs for NovA and TherA were 21.7% (10/46) and 43.5% (20/46) for ≥75, 58.7% (27/46) and 10.9% (5/46) for ≥50, and 32.6% (15/46) and 45.7% (21/46) for ≥25%, respectively.

The comparison of VenA with the other three antibodies demonstrated poor results for the overall agreement, positive and negative agreement, and Kappa values, similar to the TMA cohort. A case by case comparison showed the following: NovA showed significant difference in 17 out of 46 cases, including positive staining in VenA-negative cases; TherA differed significantly in 20/46 cases, AbcA showed a significant difference in 8/46 cases. Four positive cases were negative with VenA; one negative case was minimal positive with VenA; and three cases whose positivity was either higher or significantly lowered with VenA, compared to AbcA.

### DLL3 expression in cell cultures

**Fig. 3:**
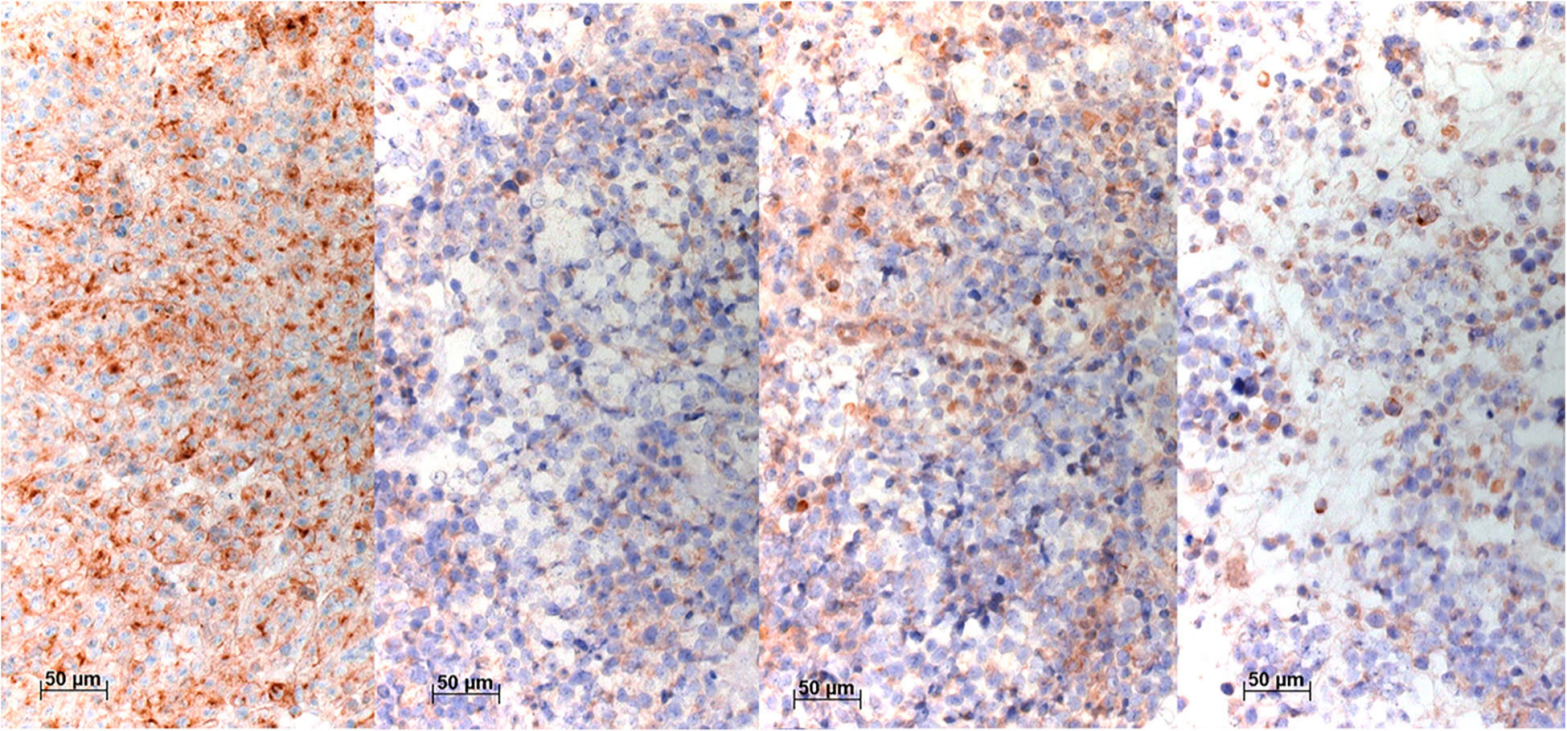
Immunocytochemical stainings of NCI-H69s with VenA, AbcA, NovA, and TherA (left to right). VenA shows an intense membranous but also a cytoplasmic staining, the latter concentrated as dots, most likely representing accumulation within endoplasmic reticulum. AbcA stained less than half of the cells, whereas NovA and TherA stained approximately 50% of the cells, but in variable intensity. Bar 50 μm

**Fig. 4:**
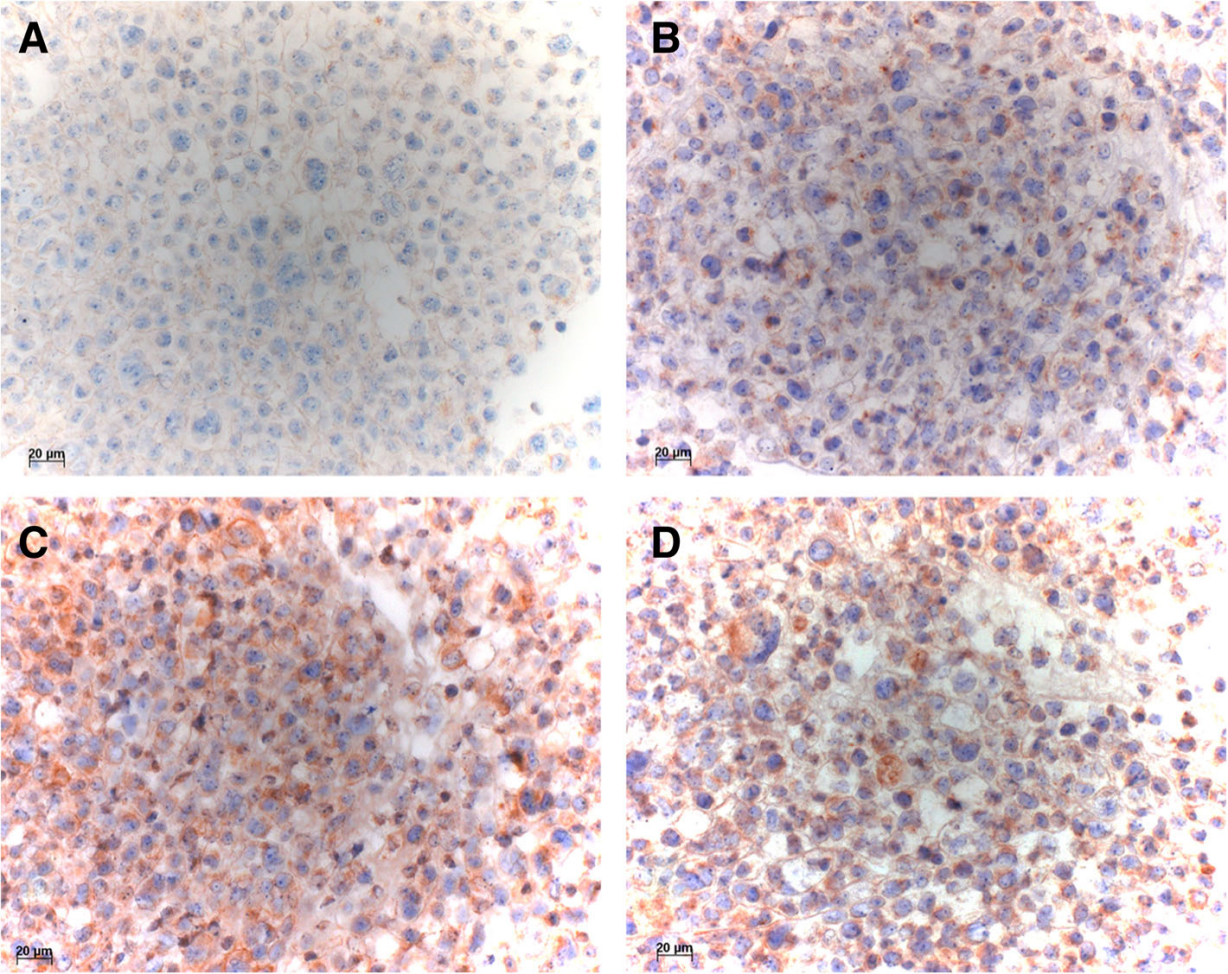
Immunocytochemical stainings of NCI-H69a with VenA (**a**), AbcA (**b**), NovA (**c**), and TherA (**d**). VenA (**a**) is completely negative in the carcinoma cells, whereas AbcA (**b**) and TherA (**d**) stained many cells, and NovA (**c**) stained almost all cells intensely. Bar 20 μm

Positive DLL3 reaction was observed in cell lines with high NE and low Notch scores (NCI-H69, NCI-H187, NCI-H740, and NCI-H735). The same was observed for all four antibodies in NCI-H69s cell line (Fig. 3). The VenA stained 90% of cells in smears, and 80% of cells in cytoblock. The AbcA stained 90% of cells in smears, and 60% of cells in cytoblock. Positivity of the cells in smears and cytoblocks was 80 and 40% for NovA, and 80 and 40% for TherA, respectively. On the other hand, when tested on the adherent NCI-H69a cell line, known to be negative for DLL3, staining with VenA showed a negative reaction in smears and in cytoblock preparations, whereas all the other antibodies showed positive reactions in different ranges (10–90% of positive cells) (Fig. 4). The cell line NCI-H1048, also known to be DLL3-negative, did not show positive reaction with VenA, whereas variable positivity was seen with other three antibodies.

### Immunohistochemical expression of *TP53* and *RB1* and correlation with DLL3

Due to the superior performance of VenA, the analyses were performed only in regards to this antibody.

No significant correlation was observed between DLL3 expression (VenA) and the expression of *p53* and *RB1* in LCNEC. Similarly, no significant correlation was observed between DLL3 expression (VenA) and the expression of *p53* in SCLC, whereas, *RB1* loss in SCLC showed statistical significant correlation with the DLL3 positivity (*p* = 0.037). p53 protein expression was present in 15/21 (71.4%) of SCLC, and in 15/28 (53.6%) of LCNEC, while 9/24 (37.5%) of SCLC and 16/27 (59.3%) of LCNEC expressed RB1.

In all performed and analyzed immunohistochemical reactions, differences in staining pattern, percentage and/or intensity in old vs more recent samples were not observed.

## Discussion

Recently, first-in-human, first-in-class, open-label phase 1 study of a DLL3-targeted antibody-drug conjugate was published [18], paving the way for the treatment of patients with SCLC, expressing DLL3 on the tumor cells. Since there has not been any significant improvement in therapy for this group of patients for many decades, and they are usually diagnosed at a later stage, this represents a tremendous step forward. Keeping this in mind, and the fact that therapy decision will be based on immunohistochemical DLL3 expression, we tested four different antibodies, VenA, NovA, TherA, and AbcA to evaluate their adequacy for this evaluation.

Using VenA (antibody used in the clinical trial) as a reference antibody, we have shown that none of the other three antibodies can reliably be used for the DLL3 test. Differences in expression with respect to VenA were observed, regardless of the cut-off values (≥75%, or ≥ 50%, or ≥ 25%), that were applied. This was true for samples from patients with SCLC as well as with LCNEC.

In our study, different results in DLL3 expression in SCLC, applying 50% as a cut-off, were obtained in study cohort and validation cohort (45.8% vs 78%, respectively). These might be best explained as the matter of sampling issue: in the study cohort, the samples from the resection material were analyzed, while in the validation cohort small biopsy samples dominated.

To evaluate the specificity of all these antibodies, we used SCLC cell cultures with known expression of neuroendocrine markers, DLL3 and Notch1/3. Testing the antibodies on these cell cultures, using smears and cytoblocks, we showed that VenA is the only antibody that reliably stains tumor cells known to be DLL3 positive, and is also negative in cells that do not express DLL3. Thus, cell lines NCI-H69a and NCI-H69s can serve as a control for other antibodies. It is known that batches of antibodies can vary quite substantially in specificity; cell blocks from these cell cultures provide reference standards. These are superior to in-house produced tissue references, as the amount of tumor tissue in SCLC biopsies is very limited.

Although LCNEC was excluded in the clinical trial due to a small sample size, we have tested its DLL3 expression in this study using a larger sample size. Approximately 50% of the tumors were positive, and the expression values (scores) were higher in several cases compared to SCLC. Therefore, DLL3-based toxin-coupled antibody treatment might be an option for these patients, if they express DLL3. Interestingly, new treatment options have been proposed for LCNEC, such as chemotherapy based on an SCLC regimen for cases with *RB1* loss and cisplatin-based chemotherapy for cases with loss of *PTEN* or activating mutation of *PI3KCA* [8, 9]. As shown here, there is no correlation between *RB1* loss and DLL3 expression in LCNEC. Therefore, anti-DLL3 therapy is another new option for these patients. This investigation fails to answer whether the positive statistical correlation between RB1 loss and DLL3 expression is a real association or only because most SCLC cases show this loss. However, as RB1-loss is typically found in neuroendocrine marker expressing SCLC this might point to the associated DLL3-expression in this set of carcinomas.

Finally, our study has several limitations. We have used two cohorts, with different sample types (resections vs small biopsies). Both cohorts were rather small. We have used archived tissue, which is not optimal for protein expression analysis, but is a real-life situation.

## Conclusions

DLL3 immunohistochemical evaluation will be mandatory for the decision about an application of the DLL3-targeted antibody-drug conjugate in patients with SCLC. The EMA, upon release of the drug, usually requires the use of a validated test. Therefore, several antibodies, if successfully validated, might be used. In this study, we have shown the superiority of the antibody used in the clinical study in detecting DLL3 expression. Furthermore, we have demonstrated that, like for other antibodies, positive and negative probes are mandatory. We have established cell cultures as test samples which can be used for further DLL3 antibodies to be tested. We have also provided evidence, that patients suffering from LCNEC express DLL3 in high proportions, which might provide basis for studies of new therapy options in this group of patients.

## Abbreviations

ASCL-1: Achaete-scute homolog 1
ATCC: American Type Culture Collection, LGC Standards GmbH, Wesel, Germany
DLL-3: Delta-like ligand 3
EMA: European Medicines Agency
LCNEC: Large cell neuroendocrine carcinoma
NE: Neuroendocrine
PD-L1: Programmed death-ligand 1
POU2F3: POU class 2 homeobox 3
SCLC: Small cell lung cancer
TherA: DLL3 antibody, PA5–26336, Thermo Fisher Scientific, Waltham, MA, USA AbcA Dll3 antibody, ab103102, Abcam, Cambridge, MA, USA
TMA: Tissue microarray
VenA: DLL3 ready-to-use assay, clone SP347, Ventana, Roche, Tucson, AZ, USA) NovA DLL3 antibody, NBP2–24669, Novus Biological, Littleton, CA, USA,

## References

[1] Sabari JK, Lok BH, Laird JH, Poirier JT, Rudin CM (2017). Unravelling the biology of SCLC: implications for therapy. Nat Rev Clin Oncol.

[2] Ferlay J, Soerjomataram I, Ervik M, et al. GLOBOCAN 2012 v1.0, Cancer Incidence and Mortality Worldwide: IARC CancerBase No. 11 [Internet]. Lyon : International Agency for Research on Cancer; 2013. Available from: http://globocan.iarc.fr, Accessed 22 Aug 2018.

[3] American Cancer Society. Global Cancer facts and figures 3rd edn, American Cancer Society 2015.

[4] Früh M, De Ruysscher D, Popat S (2013). Small-cell lung cancer (SCLC): ESMO Clinical Practice Guidelines for diagnosis, treatment and follow-up. Ann Oncol.

[5] National Comprehensive Cancer Network Guidelines Version 2.2018 Small Cell Lung Cancer. https://www.nccn.org/professionals/physician_gls/pdf/sclc.pdf Accessed 22.08.2018.

[6] von Pawel J, Jotte R, Spigel DR (2014). Randomized phase III trial of amrubicin versus topotecan as second-line treatment for patients with small-cell lung cancer. J Clin Oncol.

[7] Gazdar AF, Bunn PA, Minna JD (2017). Small-cell lung cancer: what we know, what we need to know and the path forward. Nat Rev Cancer.

[8] Karlsson A, Brunnström H, Micke P (2017). Gene Expression Profiling of Large Cell Lung Cancer Links Transcriptional Phenotypes to the New Histological WHO 2015 Classification. J Thorac Oncol.

[9] Derks JL, Leblay N, Thunnissen E (2018). Molecular Subtypes of Pulmonary Large-cell Neuroendocrine Carcinoma Predict Chemotherapy Treatment Outcome. Clin Cancer Res.

[10] Saunders LR, Bankovich AJ, Anderson WC (2015). A DLL3-targeted antibody-drug conjugate eradicates high-grade pulmonary neuroendocrine tumor-initiating cells in vivo. Sci Transl Med.

[11] Geffers I, Serth K, Chapman G (2007). Divergent functions and distinct localization of the Notch ligands DLL1 and DLL3 in vivo. J Cell Biol.

[12] Dunwoodie SL, Henrique D, Harrison SM, Beddington RS (1997). Mouse Dll3: a novel divergent Delta gene which may complement the function of other Delta homologues during early pattern formation in the mouse embryo. Development.

[13] Loomes KM, Stevens SA, O'Brien ML (2007). Dll3 and Notch1 genetic interactions model axial segmental and craniofacial malformations of human birth defects. Dev Dyn.

[14] George J, Lim JS, Jang SJ (2015). Comprehensive genomic profiles of small cell lung cancer. Nature.

[15] Jiang T, Collins BJ, Jin N (2009). Achaete-scute complex homologue 1 regulates tumor-initiating capacity in human small cell lung cancer. Cancer Res.

[16] Borromeo MD, Savage TK, Kollipara RK (2016). ASCL1 and NEUROD1 Reveal Heterogeneity in Pulmonary Neuroendocrine Tumors and Regulate Distinct Genetic Programs. Cell Rep.

[17] Huang YH, Klingbeil O, He XY (2018). POU2F3 is a master regulator of a tuft cell-like variant of small cell lung cancer. Genes Dev.

[18] Rudin CM, Pietanza MC, Bauer TM (2017). Rovalpituzumab tesirine, a DLL3-targeted antibody-drug conjugate, in recurrent small-cell lung cancer: a first-in-human, first-in-class, open-label, phase 1 study. Lancet Oncol.

[19] Tanaka K, Isse K, Fujihira T (2018). Prevalence of Delta-like protein 3 expression in patients with small cell lung cancer. Lung Cancer.

[20] Zhang W, Girard L, Zhang YA (2018). Small cell lung cancer tumors and preclinical models display heterogeneity of neuroendocrine phenotypes. Transl Lung Cancer Res.

